# Microscopic Imaging to Visualize the Distribution of Dietary Nucleic Acids in Food Products of Various Origins

**DOI:** 10.3390/foods12213942

**Published:** 2023-10-28

**Authors:** Anna Kościelak, Zuzanna Koziara, Ana Pons Maria, Rafał Płatek, Agnieszka Bartoszek

**Affiliations:** 1 Department of Food Chemistry, Technology and Biotechnology, Faculty of Chemistry, Gdańsk University of Technology, 80-233 Gdansk, Poland; anna.koscielak@pg.edu.pl (A.K.); zuzanna.koziara@pg.edu.pl (Z.K.); ana.pons.maria@gmail.com (A.P.M.); 2 Laboratory for Regenerative Biotechnology, Department of Molecular Biotechnology and Microbiology, Faculty of Chemistry, Gdańsk University of Technology, 80-233 Gdansk, Poland; rafal.platek@pg.edu.pl

**Keywords:** dietary nucleic acids, nucleic acids visualization in food, microscopic images, cryosectioning, FFPE

## Abstract

Dietary nucleic acids (dietNAs) are being increasingly recognized as important food components with nutritional value. However, the precise dietary recommendations for dietNAs are limited, because established methods for determining the quantity and nutritional role of dietNAs are still lacking. One of the tools to narrow this gap could be microscopic imaging, as a convenient approach to visualize the abundance and distribution of dietNAs in food products. With the aid of appropriate bioinformatic elaboration, such images may in future enable the direct semiquantitative estimation of these macromolecules in food products. In the presented study, two methods of preparing microscopic sections and staining them with DNA-specific fluorochromes were used for microscopic imaging of dietNAs in food products of plant and animal origin. Procedures for preparing formalin-fixed paraffin-embedded sections and cryosections were compared in terms of their usefulness for routine food analysis. Both methods turned out equally suitable for visualizing dietNA distribution in animal and plant products. However, the use of cryosections allowed a significantly shorter analysis time and reduced the consumption of organic solvents. Both of these advantages make the cryosection method preferable while establishing a dedicated methodology for routine assessment of dietNAs in the food industry.

## 1. Introduction

Nucleic acids are found in every living cell; therefore, the majority of consumed food products may be expected to be a source of dietary nucleic acids (dietNAs) in a human diet, both DNA and RNA, and/or their components. Surprisingly, nowadays, dietNAs are rarely considered chemical food components or nutritionally relevant food ingredients. As a consequence, there are no established methods for determining quantity or nutritional role of dietNAs. However, some recent publications indicate that this omission may soon be repaired [1,2]. Our former research on the role of dietNAs as a food component resulted in the development of a toolbox of methods to enable an initial characterization of the abundance and quality of dietNAs in different raw and processed foods [3]. One of the proposed approaches, which proved to be very effective for the visualization of dietNA distribution in foodstuffs, relied on the preparation of formalin-fixed paraffin-embedded (FFPE) sections of food samples stained with dedicated, DNA- or RNA-specific fluorochromes. Importantly, this method, when combined with image analysis, has potential for further evolvement into quantitative estimation of dietNA abundance. However, preparation of FFPE sections is time consuming and not environmentally friendly due to extensive use of organic solvents. It includes several days of sample fixation, formation of paraffin blocks, then cutting them and staining of obtained microscopic sections. This lengthy procedure for preparation of FFPE sections excludes this method from routine use for dietNA assessment. A convenient methodology would be necessary for the food industry if dietNAs reach nutrient status. Additionally, long procedures and the use of organic solvents may be potential sources of damage to dietNAs. In particular, it would inevitably lead to the degradation of RNA that may be contained in food samples, as it has been suggested that in some food products, RNA content is higher than DNA content. For instance, some scientific reports showed that RNA content in seafood could be more than four times the amount in DNA [4,5]. Therefore, RNA losses can cause significant misrepresentation in estimated nutritional value associated with the content of dietNAs in a given food product. 

The objective of the presented research was to compare the suitability of two established methods of tissue section preparation, FFPE and cryosectioning, for microscopic dietNA analysis. This comparison focuses on dietary DNA as a more stable food component and for whose detection a choice of selective chromophores is commercially available. To compare the discussed methods, food products representing various groups (animal, plant, and dairy products) and differing in the degree of processing were used. The analysis included a comparison of the duration of sample preparation procedures, the number of organic solvents used, the equipment needed, the usefulness of the methods in relation to various types of food products, and the quality of the obtained microscopic images. The results of the conducted research may be useful as a starting point for the development of software solutions for a semiquantitative, yet sufficient for dietary recommendations, estimation of dietNA abundance.

## 2. Materials and Methods

### 2.1. Chemicals and Biochemicals

Anhydrous ethyl alcohol, 96% (*v*/*v*) ethyl alcohol, 36–38% (*v*/*v*) formaldehyde, and xylene were purchased from POCH (Gliwice, Poland). The ethylenediaminetetraacetic acid (EDTA), Tris-HCl, dimethyl sulfoxide (DMSO), phosphate-buffered saline (PBS), glycerol, normal melting point (NMP) agarose and Neo-Mount anhydrous resin were purchased from Sigma-Aldrich (St. Louis, MO, USA). One tablet of PBS was dissolved in 200 mL of water to create a PBS solution (137 mM NaCl, 10 mM phosphate, 2.7 mM KCl, pH 7.4). In this study, histological paraffin (Histosec pastilles, Merck, Darmstadt, Germany), 4% (*v*/*v*) formaldehyde solution in phosphate buffer (Mega Herba, Blonie, Poland), glacial acetic acid (Merck, Germany), Tissue Freezing Medium (Leica Biosystems, Nussloch, Germany), food-grade gelatine (Lidl, Neckarsulm, Germany), Hoechst 33342 (working solution: 1 μL of 20 mM Hoechst 33342 in 1 mL of distilled water, Thermo Scientific, Waltham, MA, USA), SYBR Green working solution (10,000× diluted in Tris-EDTA (TE) buffer (10 mM Tris-HCl, 1-mM EDTA, pH 7.5, Sigma-Aldrich, USA), eosine (70% ethanolic solution, Serva, Heidelberg, Germany), and Delafields hematoxylin were used. Water was purified with MilliQ system (Millipore, Merck, Germany).

### 2.2. Food Samples

Pork ham was purchased from the local shop of Meat Company Nowak, Jankowo, Northern Poland. Chicken liver was derived from the farmers market, in Gdańsk, Poland. Canned green bean and mozzarella cheese were purchased from the local supermarket in the Pomerania region, Poland. All samples were kept at 4 °C until processed and were embedded in paraffin or in Tissue Freezing Medium on the same day they were bought.

### 2.3. Preparation of Paraffin-Embedded Sections of Meat and Cheese Samples

Preparation of paraffin-embedded food samples (pork ham, chicken liver, mozzarella) was performed according to the procedure described by Cieślewicz et al. [3]. Initially, from each meat or cheese sample, 5 fragments with dimensions of 0.5 × 0.5 × 1.0 cm (H × W × L) were cut out. These samples were fixed in 4% (*v*/*v*) formaldehyde in phosphate buffer, dehydrated with ethanol solutions of increasing concentration, and submerged in xylene as described previously [3]. Finally, the food samples were transferred to a liquid histological paraffin (Histosec pastilles, Merck, Germany) and thermostated at 58 °C in a drying and heating chamber (Binder GmbH, Tuttlingen, Germany), where they remained for 24 h. Molding of the paraffin blocks was performed according to the formerly described procedure [3]. The resultant paraffin blocks were cut with the manual rotary microtome RM50H (Biomed, Warszawa, Poland) into 10-μm-thick sections. The obtained paraffin-embedded tissue sections were transferred onto the glass slides, precoated with Mayer’s albumin adhesive (prepared as previously described [6]), smoothed out by adding water drops, and then left on the heated surface (37 °C) until fully dried and adhered to the glass. The resultant sections were stored at room temperature.

### 2.4. Preparation of Paraffin-Embedded Sections of Plant Samples

The preparation of paraffin-embedded plant samples (canned green beans) was performed according to the procedure described by Cieślewicz et al. [3]. Whole green bean seeds were submerged in FAA fixative (48% ethyl alcohol, 3.7% formaldehyde, 5% glacial acetic acid), dehydrated with ethanol solutions of increasing concentration, and submerged in xylene as described previously [3]. Further preparation stages were followed as described in Section 2.3.

### 2.5. Preparation of Cryosections of Food Samples

The fragments of pork ham, chicken liver, and mozzarella with dimensions of 0.5 × 0.5 × 1.0 cm (H × W × L) were cut out. All sample cuts and bean seeds were transferred to histological plastic molds previously filled with a thin layer of Tissue Freezing Medium. The freezing medium was added to fully cover the samples. The samples were immersed in liquid nitrogen for 3 min, then transferred to a freezer (−20 °C) for 20 min. The frozen blocks with the embedded sample fragments were removed from the plastic molds, accommodated in the manual cryostat CM1520 (Leica Biosystems, Germany), and cut frozen into 10-μm-thick sections at −20 °C. The obtained frozen tissue sections were transferred to the microscope glass slides (VWR, Radnor, PA, USA) covered with 0.5% (*w*/*v*) gelatine solution and left for 10 min at room temperature until sections were fully adhered to the slide’s surface. All microscope slides were stored in the fridge at 4 °C.

### 2.6. Basic Tissue Staining

The food tissue sections were stained with hematoxylin and eosin to visualize proteins and the overall tissue structures. First, the paraffin-embedded tissue sections were dewaxed and rehydrated according to the procedure described by Cieślewicz et al. [3]. Both paraffin-embedded tissue sections and frozen tissue sections were immersed in water and stained first with hematoxylin and then with 70% (*v*/*v*) ethanolic eosin solution as described by Cieślewicz et al. [3]. Each stained section was mounted with a drop of Neo-Mount resin and covered with a cover slip. The sections were left overnight at room temperature and then analyzed using a light Olympus BX 60 microscope (Evident, Waltham, MA, USA) applying ×200 magnification.

### 2.7. Fluorescent Staining of Nucleic Acids

The food tissue sections were stained with DNA-selective fluorescent dyes Hoechst 33342 or SYBR Green which enabled the staining of deoxyribonucleic acid in blue or green, respectively. The paraffin-embedded tissue sections were dewaxed and rehydrated as described in Section 2.6. Both paraffin-embedded tissue sections and frozen tissue sections were shortly rinsed with distilled water. Then, 1–2 drops of appropriate dye working solution were applied onto the surface of prepared tissue sections for 10 and 20 min for Hoechst 33342 and SYBR Green, respectively. During staining, the slides were protected from light. After staining, the slides were washed in deionized water for 1 min, mounted with 90% (*v*/*v*) glycerol in PBS solution, and covered with a cover slip. All slides were stored in a fridge (4 °C) for 24 h, and then they were analyzed under fluorescent light using an Olympus BX 60 microscope (Evident, USA) applying ×200 magnification.

## 3. Results

**Figure 1:**
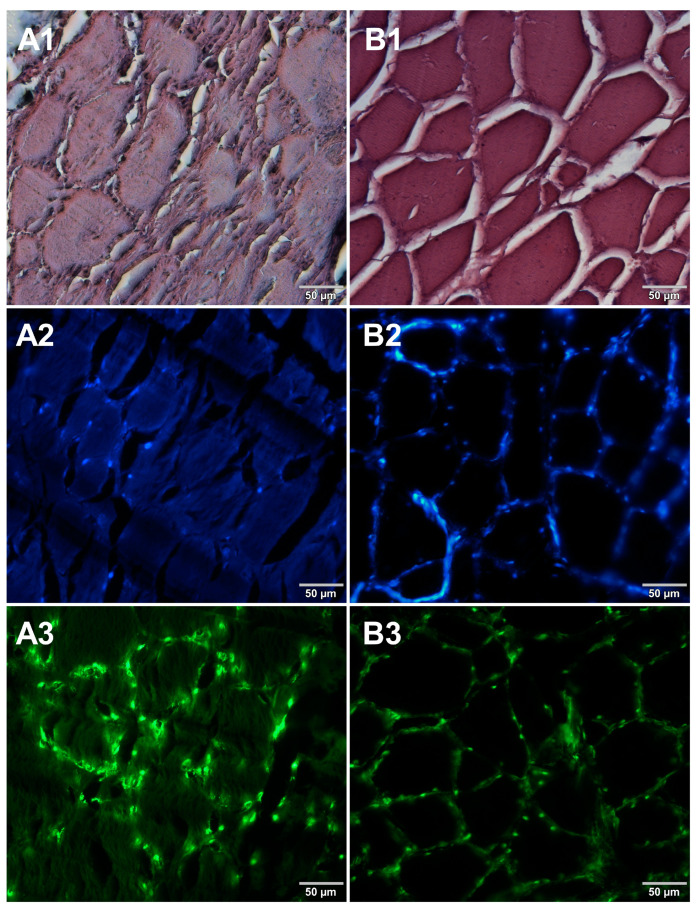
Comparison of methods for preparing microscope sections from cured pork ham samples. Microscopic images are representative of either paraffin-embedded samples (**A1**–**A3**) or cryosections (**B1**–**B3**) stained with 1—eosin and haematoxylin, 2—Hoechst 33342, or 3—SYBR Green. All images were recorded at a magnification of 200×.

**Figure 2:**
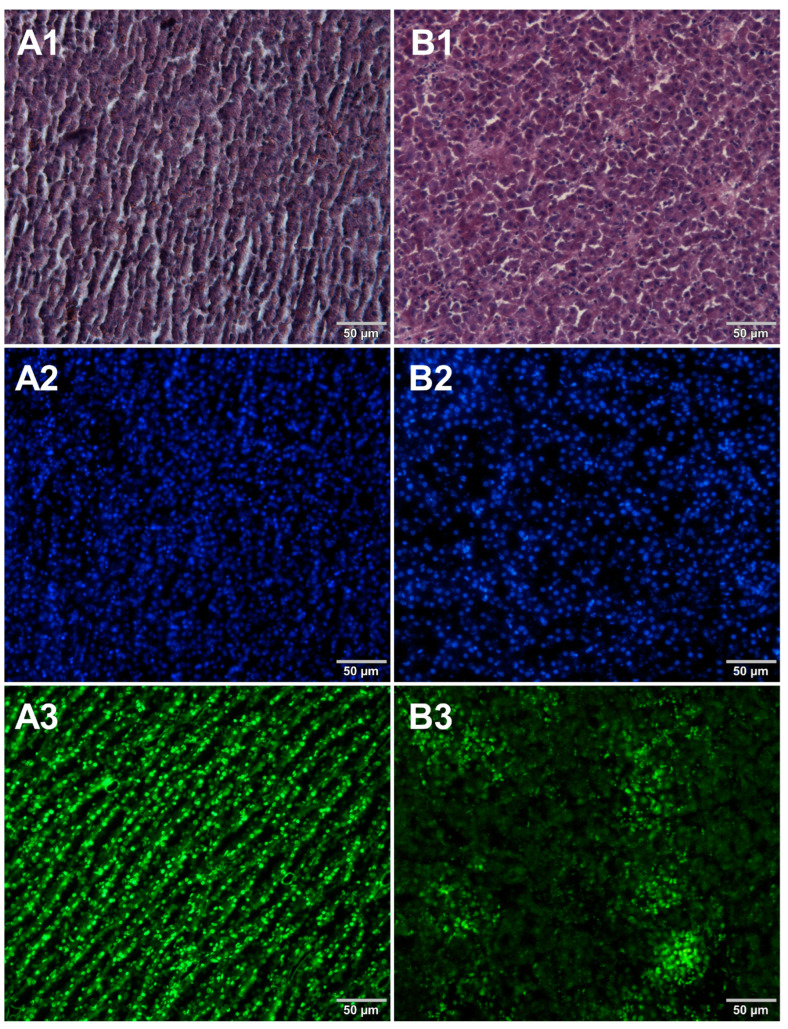
Comparison of methods for preparing microscope sections from raw chicken liver samples. Microscopic images are representative of either paraffin-embedded samples (**A1**–**A3**) or cryosections (**B1**–**B3**) stained with 1—eosin and haematoxylin, 2—Hoechst 33342, or 3—SYBR Green. All images were recorded at a magnification of 200×.

**Figure 3:**
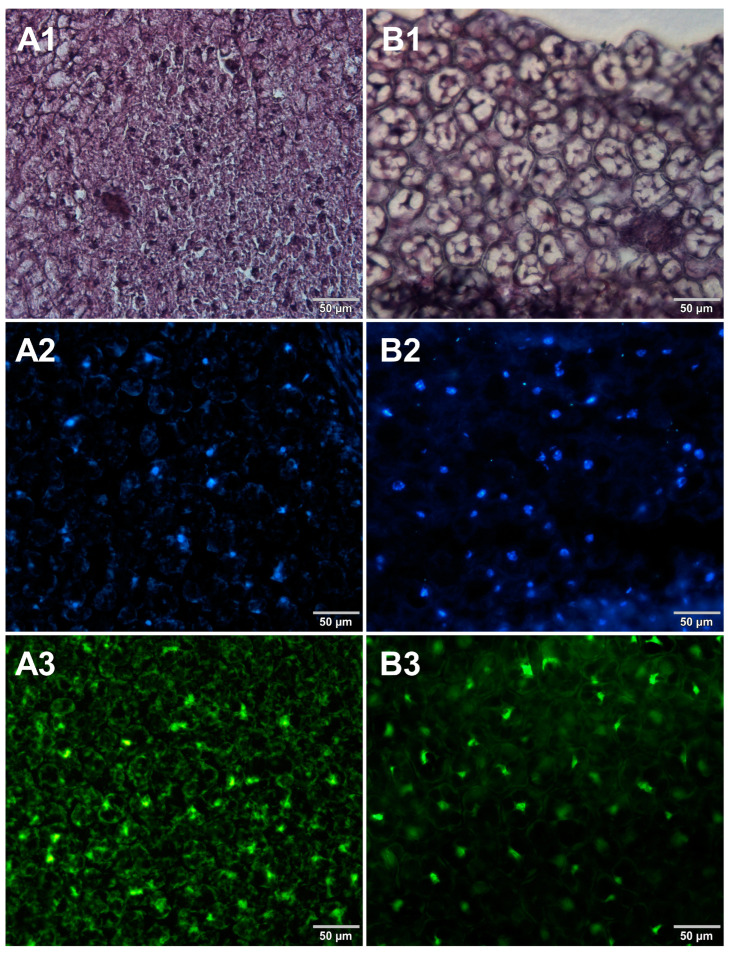
Comparison of methods for preparing microscope sections from canned bean samples. Microscopic images are representative of either paraffin-embedded samples (**A1**–**A3**) or cryosections (**B1**–**B3**) stained with 1—eosin and haematoxylin, 2—Hoechst 33342, or 3—SYBR Green. All images were recorded at a magnification of 200×.

**Figure 4:**
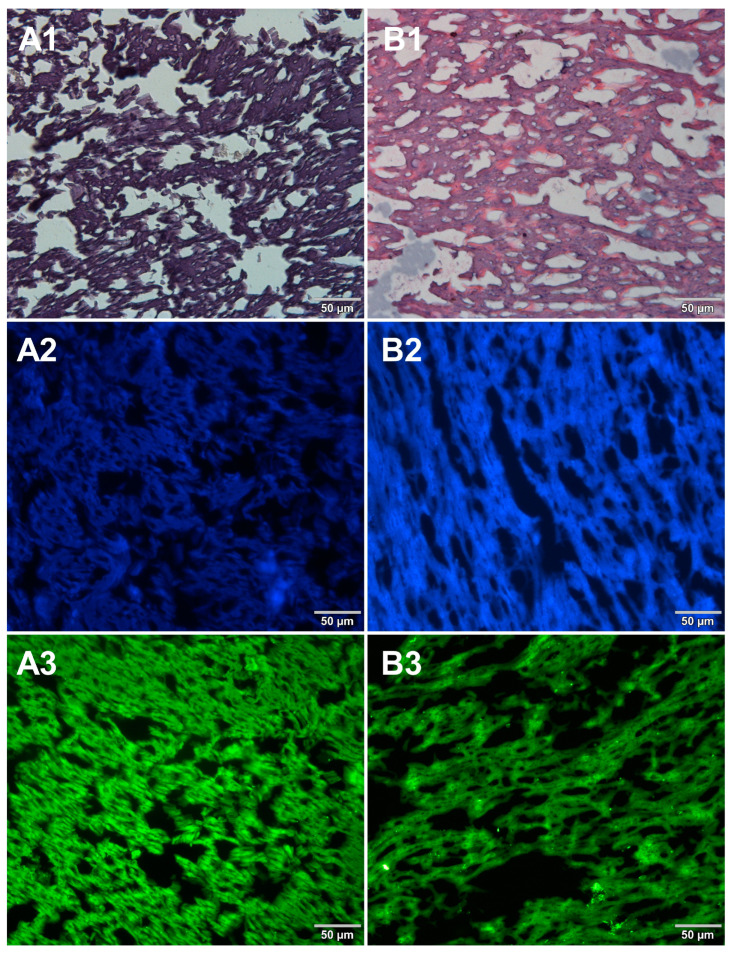
Comparison of methods for preparing microscope sections from mozzarella cheese samples. Microscopic images are representative of either paraffin-embedded samples (**A1**–**A3**) or cryosections (**B1**–**B3**) stained with 1—eosin and haematoxylin, 2—Hoechst 33342, or 3—SYBR Green. All images were recorded at a magnification of 200×.

Microscopic sections of various food products were prepared using paraffin-embedding and cryosectioning, both of which are well-established histological techniques. The obtained sections transferred onto microscopic slides were stained with either basic staining technique (Figure 1, Figure 2, Figure 3 and Figure 4, panels 1) or with the use of DNA-specific fluorochromes (Figure 1, Figure 2, Figure 3 and Figure 4, panels 2 and 3). Basic staining was applied to provide a general overview of tissue structures in studied food samples as well as information on protein abundance. The purplish-blue haematoxylin stains cell nuclei, while reddish-pink eosin stains cytoplasm and other cellular protein structures. The dedicated fluorochromes were applied to visualize DNA distribution in the food tissue sections. Hoechst 33342 stains nuclear DNA in blue and SYBR Green in green. The comparisons of the resultant representative microscopic images of stained sections of studied foodstuffs obtained with the mentioned techniques are presented in Figure 1, Figure 2, Figure 3 and Figure 4.

Both methods of the preparation of sections from the tested food products have proven successful, while the quality of the images was similar. Tissue integrity was maintained for both meat food products such as cured pork ham (Figure 1) or raw chicken liver (Figure 2) as well as for the processed plant products such as canned green beans (Figure 3). Only in the case of FFPE sections, the fixation procedure with the use of organic solvents could also cause a slight tearing of the pork ham tissues (Figure 1), which was particularly visible in the case of the basic staining with eosin and hematoxylin. Cell nuclei in ham (Figure 1), liver (Figure 2), and bean (Figure 3) samples are clearly visible in microscopic images of sections stained with fluorochromes. In the case of mozzarella cheese sections, more differences in the images were observed (Figure 4), probably due to heterogeneity of this foodstuff. The images obtained for mozzarella cheese (Figure 4) indicate the presence of nucleic acids in a dispersed form, the source of which may be both milk and microorganisms. For instance, nucleic acids may be released from milk somatic cells degraded during fermentation stage in the production of this type of food. The detected intact cells appear larger in cryosection than FFPE images, which may be due to the preparation steps where frozen sections were not dehydrated; in the case of paraffin-embedded tissues, the cells’ structure may be affected by the loss of water.

**Figure 5:**
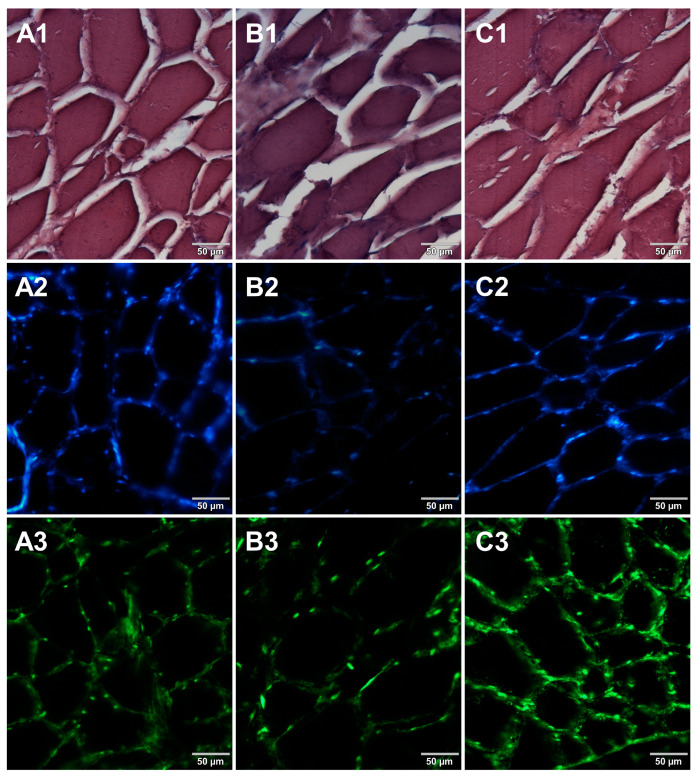
Comparison of different types of microscope slides. Microscopic images of cryosections of cured pork ham samples that were prepared on slides precoated with (**A1**–**A3**) gelatin, (**B1**–**B3**) low melting point agarose, or (**C1**–**C3**) on adhesive slides. The samples were stained with 1—eosin and haematoxylin, 2—Hoechst 33342, or 3—SYBR Green. All images were recorded at a magnification of 200×.

**Figure 6:**
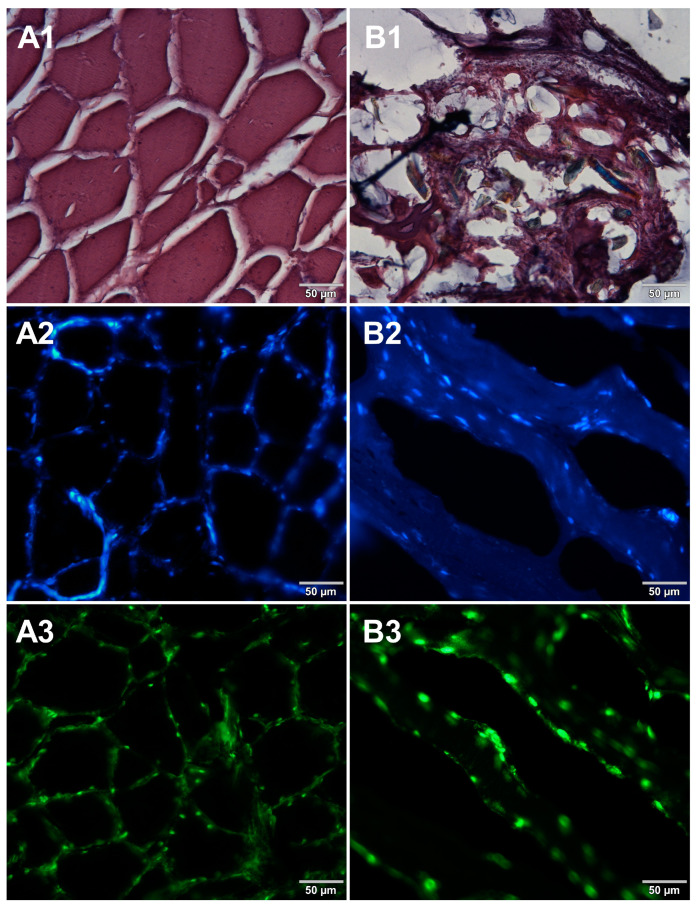
Comparison of freezing methods. Microscopic images of cryosections of cured pork ham samples that were frozen in (**A1**–**A3**) liquid nitrogen and (**B1**–**B3**) at −20 °C. The samples were stained with 1—eosin and haematoxylin, 2—Hoechst 33342, or 3—SYBR Green. All images were recorded at a magnification of 200×.

In the course of initial experiments, some technical obstacles were encountered during the preparation of frozen sections due to the poor adhesion of tissue sections to the glass surface of microscope slides. To overcome this problem, three types of microscope slides with different surfaces were compared. Figure 5 shows microscopic images of cured pork ham sections adhered to microscope slides coated with either 0.5% (*w*/*v*) gelatin (Figure 5A1–A3) or 0.5% (*w*/*v*) agarose (Figure 5B1–B3) or to commercially available adhesive slides with a positively charged surface, which helps to bond tissue sections (Leica Biosystems, Germany) (Figure 5C1–C3). For gelatin-coated and adhesive slides, no significant differences were observed between the obtained images. In the case of agarose-coated slides, slight damage to the tissue structure was seen. Although the use of gelatin to increase the adhesion of the surface of the slides requires additional preparation time, it is much less expensive than the commercially available adhesive slides. Therefore, gelatin-coated slides were selected for further preparations of frozen sections of food products. In the next step, two methods of freezing during the formation of frozen blocks were compared. Microscopic images obtained for samples frozen by both methods are shown in Figure 6. Freezing at −20 °C resulted in severe damage of the sample structure. The tearing of the tissue caused the appearance of large spaces between cells (Figure 6B1–B3) due to the formation of ice crystals. Quick freezing with liquid nitrogen prevented the formation of crystals, keeping the tissues intact (Figure 6A1–A3). The described observations were taken into account when establishing the final procedure of the preparation of cryosections of food samples described in Section 2.5.

## 4. Discussion

The results presented in Figure 1, Figure 2, Figure 3 and Figure 4 confirm that both methods used, i.e., cryosectioning and FFPE, are suitable for visualizing the abundance and distribution of dietNAs in food products of various origins. Nonetheless, certain differences in the procedures used to prepare paraffin-embedded sections and cryosections may affect their suitability for routine food analysis. A comparison of these two methods, broken down according to analysis steps, is shown in Table 1. Both the literature reports and our own experimental observations were used to assemble this comparison. In the case of paraffin embedding, the multistage tissue fixation step prolongs the duration of the whole procedure. It also involves the use of various types of organic solvents, such as ethanol or xylene, which can accelerate the degradation of RNA contained in food samples and are not indifferent to human health and the environment. The cryosectioning technique, due to the omission of the tissue fixation step, enables the preparation of frozen microscopic sections in a very short time. However, an inconvenience of this latter technique is the formation of ice crystals within the sample structure during the formation of frozen blocks. There are several approaches to avoid this drawback. One of the solutions applied in our study to reduce the occurrence of ice crystals was the use of liquid nitrogen for tissue block freezing. It is also possible to use other cooling media, such as isopentane, which provides better penetration of cold into the tissue. Another suggested way to prevent the formation of ice crystals is to saturate the samples with a sucrose solution before freezing [7]. However, this step extends the duration of the whole procedure, which is why the first option was chosen in this study.

Blocks forming is the next step in preparation of food sections. In the case of cryosectioning technique, solidification of food sample embedded in freezing medium in liquid nitrogen takes 3 min, followed by 20 min in a freezer (−20 °C), and does not require prior preparation of the embedding medium. In the case of FFPE technique, block forming lasts longer. Liquid paraffin must be thermostated and deaerated before molding. Its solidification takes at least an hour, depending on whether paraffin-embedded samples in molds are left on ice or at room temperature. The virtue of the latter technique is that once paraffin blocks are prepared, it is possible to store solidified food samples in that way for a very long time. Storing food samples in solidified blocks may be useful when further steps of dietNA analysis are outsourced by the food industry to an external laboratory. Although the advantage of FFPE technique is the ability to store paraffin blocks and sections for many years at room temperature, research shows that such prolonged storage increases the damage of nucleic acids present in the samples [10]. In the case of food samples immersed in a freezing medium, the frozen microscope sections require only short-term storage at −80 °C, which allows the properties of the food sample to be preserved for further analysis. 

The step that requires the most specialized equipment for both techniques is the cutting of appropriately thin sections with a microtome. The microtome used for cryosectioning is typically integrated with a cryostat, making this technique more energy intensive, but again with the advantage of being less time consuming. The frozen sections laid on slides can be either stored or stained immediately after sectioning. Paraffin-embedded sections on slides should be placed on the hot plate of the section dryer (37 °C) immediately after sectioning and allowed to dry completely while adhering to the glass. Microscope slides used in both techniques gain adhesiveness if they are precoated before applying a sample section onto them. The examples of coating media are given in Table 1. Before staining with water-soluble dyes, paraffin-embedded sections need to be dewaxed and rehydrated. This extends the entire procedure by additional 20–30 min and is carried out with the use of organic solvents, e.g., xylene. In general, the selection of cryosections enables microscopic analysis to be performed within hours, while the entire procedure for paraffin-embedded sections takes several days. This is a key advantage of cryosectioning in the further development of a new potential method enabling quantitative estimation of dietNA abundance.

Although microscopic imaging is not widely used for the quantitative analysis of nutrients in food, for other applications it has been already recognized. Most often, microscopic techniques are used to study the structure of both raw and processed food products. Light microscopy and scanning electron microscopy are used to study the structural parameters of agricultural raw materials [11] as well as to follow structural changes of food matrix during and after processing [12,13,14]. In food research, paraffin-embedded sections make it possible, for example, to distinguish between fresh and frozen fish meat [15], while frozen sections are used in the imaging of starch and lipids in food products [16] or for the detection of microorganisms in food [17]. However, paraffin embedding and cryosectioning are still much better known for their use in immunohistochemistry [18,19,20,21], where these techniques are also readily explored for quantitation of some observed changes occurring in tissues [22]. Therefore, one can presume that there are potential applications for these techniques in food research. One example of such a promising application is the suggested use of microscopic imaging to estimate dietNA abundance in food samples.

## 5. Conclusions

DietNAs are food components that are gaining researchers’ attention not only because of their nutritional value, which has been already utilized by manufacturers of dietary supplements [23,24], but also because of the potential risks associated with excessive consumption of modified dietNAs contained in processed food [25]. The knowledge of dietNA content and distribution in various food products is necessary information from the perspective of research on their nutritional properties. Studies on digestion show that the distribution of nutrients in the structure of a food product affects the dynamics of their digestion and their subsequent bioavailability in the intestine [26,27,28]. As suggested here, with further development, microscopic techniques can be potentially used also to quantify dietNAs. This could be achieved by the development of a dedicated software for quantifying dietNAs based on the intensity of the light emitted by the fluorochromes used for imaging of nucleic acids in prepared microscopic sections. Such an approach to the quantification of nucleic acids for the purpose of clinicopathological diagnosis was proposed by Liu et al. [29], where ratiometric fluorescence imaging of their distribution in human tissue sections was applied.

Despite the fact that research on dietNAs is gaining momentum, there are no established methods for determining their quantity in food products. The quantitative data cited in most available publications date back to the former century [30,31]. The methods used for the quantitative determination of nucleic acids, such as cytometry [32], mass spectrometry [33], and quantitative PCR [34], are not suitable for routine use in the food industry. The most commonly used spectrophotometric method is susceptible to interference of other components, such as proteins or organic solvents [35]. Moreover, each of the mentioned quantification techniques is highly dependent on the accuracy of the procedure of nucleic acids isolation from the food product. According to research published by Cieślewicz et al. [3], the amount of nucleic acids isolated from food varies depending on the method applied, such as the use of commercial isolation kits or isolation with organic solvents. Therefore, the search for alternative ways to reliably quantify dietNAs is highly recommended.

As shown by our study, FFPE sections and cryosections are suitable for the purpose of visualization of dietNA distribution in both animal and plant products. Compared to FFPE technique, for routine analysis, cryosectioning may be recommended due to its shorter and simpler procedure. However, visualizing dietNAs provides valuable information not only on their distribution in foodstuffs, but in further perspective, has the potential to be used for the quantification of their content.

## Figures and Tables

**Table 1:** Comparison of paraffin embedding and cryosectioning methods as initial steps of dietNA determination in food products (prepared based on [3,8,9]).

Stage of dietNA Analysis	Parameter	Paraffin-Embedding	Cryosectioning
Sample fixation	Duration time	2–4 days	-
	Fixative and solvents	Formaldehyde, xylene, ethanol	Optional saturation with sucrose solution
Blocks forming	Duration time	>1 h (depending on the number of samples)	<1 h (depending on the number of samples)
	Molds	Metal or plastic	Plastic
	Embedding medium	Soft or hard paraffin wax	Freezing medium such as optimal cutting temperature (OCT) compound
	Solidification	Paraffin-embedded samples in molds left on ice	Freezing samples in molds in liquid nitrogen
	Additional equipment	Thermostat for paraffin wax	-
Sections cutting	Duration time	>1 h (depending on the number of samples)	<1 h (depending on the number of samples)
	Cutting device	Microtome	Cryostat with an integrated microtome
	Cutting temperature	Room temperature	−20 °C (but depends on the type of tissue)
	Sections storage temperature	Room temperature	−80 °C
	Slides coating	Mayer’s albumin adhesive	Poly-L-lysine, gelatin, or agarose
	Additional equipment	Section dryer hot plate	-
Staining	Duration time	>1 h (depending on the number of samples)	<1 h (depending on the number of samples)
	Dewaxing and rehydrating	Required	-

## Data Availability

The data used to support the findings of this study can be made available by the corresponding author upon request.
